# The Emergence of Artificial Intelligence-Guided Karyotyping: A Review and Reflection

**DOI:** 10.3390/genes16060685

**Published:** 2025-05-31

**Authors:** Lynne S. Rosenblum, Julia Holmes, Agshin F. Taghiyev

**Affiliations:** 1 Department of Pathology, Wake Forest University School of Medicine, Winston-Salem, NC 27157, USA; juliah@spectral-imaging.com (J.H.); ataghiye@wakehealth.edu (A.F.T.); 2 Applied Spectral Imaging, Carlsbad, CA 92009, USA

**Keywords:** artificial intelligence, chromosome analysis, clinical cytogenetics, digital karyotyping, laboratory genetics

## Abstract

Artificial intelligence (AI) has entered the medical subspecialty of cytogenetics with the recent introduction of AI-guided karyotyping into the clinical laboratory. Karyotyping is an essential component of the cytogenetic analysis process; however, it is both labor-intensive and time-consuming. The introduction of AI algorithms into karyotyping software streamlines this process to provide accurate and abundant auto-karyotyped images for laboratory professionals to review and, also, alters the paradigm for chromosome analysis. Herein, we provide an overview of the AI-guided karyotyping products currently available for clinical use, discuss their utilization in the cytogenetics laboratory, and highlight changes AI-guided karyotyping has brought for early users. Finally, we reflect on our own laboratory observations and experience to discuss issues and practices that may need to adapt to best utilize this promising new technology.

## 1. Introduction

Artificial intelligence (AI) is rapidly transforming daily life with application in areas including, but not limited to, communication, marketing, education, manufacturing, and healthcare [1]. This is evident in the medical subspeciality of cytogenetics, where AI-guided applications are entering the clinical laboratory to transform labor-intensive and time-consuming tasks, such as karyotyping, to decrease testing turn-around times, increase diagnostic yield, and improve the quality of patient care [2,3,4]. Although the potential is significant for AI to revolutionize the practice of clinical cytogenetics and chromosome analysis specifically, to date, there is a scarcity of publication on this topic. To begin to address this need, we explore this emerging technology and its integration into clinical cytogenetics laboratory practice.

Cytogenetics is a branch of cell biology and genetics which focuses on the study of the structure, function, and abnormalities of chromosomes as they relate to cell behavior during mitosis and meiosis, and in relation to the organism’s phenotype [5]. In the context of medical genetics, chromosome analysis is performed through the laboratory process of karyotyping, which allows visualization and analysis of the entire chromosome complement, and thus genome, of an individual. During this process, metaphase chromosomes derived from individual cells are identified, segmented to isolate individual chromosomes, contoured to delineate chromosome boundaries, and arranged in numerical order in a pictorial karyogram. This facilitates analysis to identify abnormalities associated with constitutional disorders and cancer [6]. Accordingly, a karyotype is defined as “the general appearance, including size, number, and shape, of the set of somatic chromosomes” [7].

Clinically, chromosome analysis has been and continues to be instrumental in a diagnostic workup in multiple fields of medicine, albeit often in conjunction with other testing modalities not addressed here [8,9]. It provides prenatal and postnatal identification of chromosomal abnormalities that cause genetic conditions, such as trisomy 21 or microdeletion syndromes. Postnatally, karyotyping may aid in the investigation of numerous conditions, including intellectual disability, short stature, recurrent miscarriage, and infertility [9,10]. In oncology, diagnosis, classification, treatment, prognosis, and disease monitoring often rely on cytogenetic testing [11]. Moreover, chromosome analysis can identify mosaicism for a constitutional chromosome abnormality and can follow clonal evolution in cancer [11] due to its focus on multiple individual metaphase cells.

The process of karyotyping has evolved from the rudimentary viewing of solid-stained chromosomes by light microscopy in the mid-twentieth century [12] to current automated cell scanning and image capture with AI-guided karyotyping applications. Here, we provide a brief history of cytogenetics and the evolution of karyotyping practice from the early days of discovery through manual laboratory processes to the current implementation of AI-guided karyotyping. We provide an overview of the AI-guided karyotyping products currently available for clinical (and research) use, discuss their utilization in the cytogenetics laboratory, and share changes AI-guided karyotyping has brought to early users. Finally, we reflect on our laboratory observations and experience, and that of others, to discuss concerns and suggest areas of practice that may need to adapt to best utilize the emerging technology.

## 2. History of Karyotyping

### 2.1. Early History

In order to appreciate the laboratory practice of karyotyping, it is necessary to understand the early history of cytogenetics and the events that lead to the emergence of the field of clinical cytogenetics. These events have been described in detail elsewhere [12,13,14,15]. Briefly, the study of human chromosomes began in the mid-20th century, but prior to the 1970s, cytogenetic analysis was performed on monochromatic stained metaphase chromosomes, which did not allow precise identification of individual chromosomes or most structural abnormalities. A major innovation occurred with the introduction of Q-banding, the first technique for identifying chromosomes by a banding pattern [16], and then additional banding techniques, such as C-, R-, NOR-, and G-banding, were developed [17]. Today, the most commonly used banding technique in somatic and constitutional cytogenetics uses trypsin digested and G-banded (Giemsa or Giemsa–Wright stained) chromosomes.

Distinct patterns of Giemsa-dark and -light bands observed on chromosomes reflect regional differences in chromatin higher-order structures and functions. These patterns are unique to each chromosome pair, and analysis of the patterns in each metaphase cell allows identification of both numerical and structural aberrations [18]. Chromosomal rearrangements, including translocations, deletions, insertions, and inversions, can be better identified now, and their correlation with cancer and constitutional disorders can be correlated.

### 2.2. Chromosome Organization and Nomenclature

To analyze metaphase chromosomes for aberrations, it was helpful to organize them visually in a logical manner in pairs rather than viewing them as a metaphase spread. Karyotyping, the process of arranging the chromosomes, and the visual representation of this arrangement as a karyogram fulfilled this function. Initially, in 1960, the Denver Conference proposed a classification system that numbered the chromosomes and placed them into seven groups (A–G) based on their length, the centromere index, and relative position of the centromere. A rudimentary nomenclature system was also proposed [19]. This classification system was subsequently revised and, in 1978, evolved into publishing the first International System for Human Cytogenomic Nomenclature (ISCN), which provided a detailed set of rules for organizing the karyogram and naming the findings in the karyotype. This document provided unifying guidelines for reporting cytogenetic findings, served and continues to serve as the central reference for cytogenetics, and is updated on a regular basis [20].

### 2.3. Manual Preparation of the Karyogram

Prior to the 1990s, preparing a karyogram involved a laborious and time-consuming process. Stepwise, metaphase cells were identified under a light microscope and the images were captured on film using an attached camera. Then, in a dark room, the film was developed, and the images were printed on photographic paper. Subsequently, double-sided tape was applied to the back of the photographs, representing each cell of interest. The individual chromosome images were cut out with scissors, and each chromosome pair was placed in its appropriate position on a paper or card stock template for analysis to create a permanent record of the cytogenetic result. This process required approximately half an hour to prepare each karyogram [21].

### 2.4. Automated Karyotyping

Improvements to the karyotyping process were developed in the late 20th and early 21st centuries with automation, which allowed for digital capture and analysis of the chromosomes using static algorithms based on incorporated data and predefined formulas [22]. During this time, roughly a dozen companies developed and marketed automated or semi-automated karyotyping systems that used computer-aided software to integrate metaphase-finding, image capture, and processing [21]. Specifically, automated karyotyping involved the processes of metaphase identification and image capture, chromosome segmentation, chromosome medial axis identification, extraction and normalization of chromosome features, and classification of chromosomes for correct karyogram placement [21,22,23,24].

The development of automated imaging and karyotyping applications faced significant challenges with metaphase and chromosome presentation. First, the captured metaphase images contain randomly placed chromosomes and, due to metaphase spreading, some chromosomes may be outside the image field. Second, the chromosomes are not rigid and linear; they can be bent, touching, and/or overlapping to form clusters. Third, there is variability in chromosome length, banding pattern, centromere index, and general morphology. Finally, the images may contain artifacts that can be inaccurately identified as chromosomes [22,24]. Thus, the extraction and computation of the chromosome image features had to account for these factors.

After decades of research and development and the use of multiple different methods and algorithms, automated karyotyping continues to have difficulties achieving fully accurate feature extraction and classification [21,22,24]. Therefore, karyograms generated by these applications require a combined approach integrating significant human intervention for corrections. Chromosome segmentation and classification largely depended on manually selecting metaphase chromosome images for automated processing; however, this is a time-consuming process subject to human error and/or bias. There is a strong need to automate the process of metaphase chromosome image selection prior to segmentation and classification of the chromosomes. Methods for categorizing metaphase chromosome images into analyzable and non-analyzable classes, with some attempt at a ranked-scale system, have been developed by a limited number of researchers [22]. Automating metaphase image selection offers two key advantages: reducing manual image selection time and streamlining image analysis. Given the current cytogenetic laboratory test volumes and staffing constraints [25], fully automated and accurate metaphase selection and karyotyping are highly desired.

## 3. AI-Guided Karyotyping

In clinical cytogenetics, AI-guided karyotyping based on machine-learning and deep-learning algorithms is poised to transform the traditional laboratory process [3]. This technology addresses the limitations of traditional automated karyotyping with systems that streamline and standardize the image capture and analysis process with high efficiency based on the extensive dataset training of algorithms. By automating the current manual processes, experts can dedicate their time to more intricate analytical tasks.

In recent years, several companies have developed AI-guided karyotyping systems that promise to revolutionize classical cytogenetics by utilizing deep learning to provide highly accurate automation for the image acquisition, segmentation, classification, and analysis of chromosomes. Currently, there are four commercially available AI-based chromosome analysis systems, from Applied Spectral Imaging^®^ (ASI, Applied Spectral Imaging, Carlsbad, CA, USA), BioView^®^ (BioView Inc., Billerica, MA, USA), Diagens^®^ Diagens (Hangzhou Diagens Biotechnology Co., Ltd., Hangzhou, Zhejiang Province China), and MetaSystems^®^ (MetaSystems Group, Inc., Medford, MA, USA) [26,27,28,29,30,31,32,33]. Here, we provide an overview and comparison of these systems based on the publicly available information from each manufacturer (Table 1), along with metrics for ASI and MetaSystems for normal blood and bone marrow specimens (Table 2).

ASI (Applied Spectral Imaging, Carlsbad, CA, USA) provides HiBand version 8.4, an AI-based chromosome analysis product that uses convolutional neural network (CNN) that is designed to learn how to automate chromosome identification, pairing, and arrangement into a karyogram. The application supports G-, Q-, and R-banding techniques and integrates with laboratory information system (LIS) and electronic medical record (EMR) software, allowing seamless data transfer. A major advantage of HiBand version 8.4 is its ability to import metaphase images captured on third-party scanning systems and generate karyograms, distinguishing it from other platforms that require proprietary data [38]. Additionally, the AI algorithm and real-time karyotype tools enhance efficiency and were shown to reduce case analysis time in normal blood and bone marrow samples by an average of 53% and 46%, respectively, during beta-testing studies of 20-cell cases [35,36]. However, there is limited data regarding the algorithm’s functionality regarding additional specimen types and classification of structurally abnormal chromosomes. Expanding the algorithm to cover a broader range of specimen types and for abnormality identification could further enhance the diagnostic utility of this platform [36,37].

The AI Karyotyping Application Suite from BioView (BioView Inc., Billerica, MA, USA) employs a CNN-based algorithm that offers chromosome identification and karyotyping with real-time karyotype correction, high-definition imaging, and abnormality identification capability. The algorithm works with BioView’s proprietary software, SoloWeb and CytoCloud, to provide secure web-based or cloud-based platforms, respectively, for remote review and analysis of scanned and AI-karyotyped cases. The web application also provides direct LIS integration of cases and legacy images. Another significant strength is its ISCN suggestion tool, which aids in generating standard nomenclature for reports. However, it is limited to G-banded chromosomes and, although images from prior analyses can be imported into a current case, it does not support active analysis of third-party metaphase images. Additionally, there is limited availability of this product for clinical use; for example, it is expressly advertised for research use only in the United States (US). Possible improvements could include training the algorithm on additional banding techniques and imported images and expansion of clinical use into additional markets [28,29].

The Diagens (Hangzhou Diagens Biotechnology Co., Ltd., Hangzhou, Zhejiang Province China) AutoVision AI-based karyotyping system utilizes CNN architecture for chromosome segmentation and identification, with an advertised greater than 99% accuracy [31]. The algorithm supports analysis of blood and prenatal specimens and G- and R-banding techniques. Uniquely, the software provides an anomaly assistance analysis function specializing in structural chromosome abnormality recognition. The AutoVision AI version 2.0 software operates exclusively with Diagen’s MetaSight scanning instrument, which allows for high-capacity (200 slides) imaging, a feature beneficial for large cytogenetics laboratories [30]. Remote access is available but limited to cloud-based connectivity, which may restrict accessibility in certain settings. Additionally, details on integration with LIS and EMR software are unavailable, but the absence of such integration would potentially impact workflow use. Potential improvements for AutoVision could include web-based access, compatibility with third-party scanning instruments, and expansion from its current restricted market into global availability [39].

The MetaSystems (MetaSystems Group, Inc., Medford, MA, USA) AI-based chromosome analysis system, Ikaros version 6.3, uses deep neural networks (DNNs) for highly accurate and reliable chromosome identification, segmentation, and classification, with a reported accuracy of 97% across specimen types in one study [40]. It supports G-, Q-, and R-banding techniques and multiple tissue types, including peripheral blood, bone marrow, amniotic fluid, and chorionic villi. The Ikaros software optimally pairs with MetaSystems’ Metafer scanning platform for image acquisition to automate workflow and efficiently produce a karyogram. Using this system, one early study found that the DNN classifier reduced technologist analysis time on average by 53.9% and 42.2% for normal blood and bone marrow 20-cell cases, respectively [37]. There is limited available data regarding the algorithm’s functionality for classification of abnormal chromosomes. Additional features of this AI-guided karyotyping product include compatibility with both automatic and manual image capture as well as capability to integrate with Neon, MetaSystems’ general data management software. Future improvements could include expanding instrument and software pairing options to include outside vendors and enhancing the algorithm for improved abnormal chromosome identification [35,40,41,42].

These AI-guided chromosome analysis systems share many similarities but also have features which make them unique (Table 1). Each employs a deep-learning approach, utilizing convolutional or deep-network architecture and a from-scratch training model requiring a large dataset rather than transfer learning that leverages information from previously trained models [3]. All the systems promise expedited image capture and more accurate chromosome analysis than previous automated (non-AI) processes, with associated improved testing quality and turn-around times. Comparison of the two systems where published data are available shows complete analysis time per metaphase cell or case, including manual corrections, and decrease in analysis time relative to the previous automated method were similar (Table 2) [35,36,37]. All four systems provide high-definition imaging, real-time tools for karyotype correction, and employ an abnormality identification algorithm. The reliance on sophisticated algorithms, the need for expert technical support, and the high financial cost of implementation are disadvantages common to all systems. There are differences among the vendors in multiple areas, such as supported specimen types and banding techniques, compatibility with scanning systems from other vendors, ease of integration with LIS and EMR software, remote access options, and markets in which they are available for clinical use. As this technology matures and is integrated into the clinical cytogenetics laboratory, it will be interesting to see which features are most valued and which become standard offerings.

## 4. Clinical Use of AI-Guided Karyotyping

AI-guided karyotyping could revolutionize cytogenetic diagnostics by combining the precision of machine learning with the power of human expertise. There have been several studies evaluating the effectiveness and efficiency of using AI-based applications for chromosome analysis either for discovery or as a precursor to clinical use. For example, to assess its accuracy, Al-Kharraz et al. [3] used a stepwise deep-learning method to automate chromosome classification of two publicly available datasets containing images of 2454 chromosomes in total. The authors achieved a combined classification accuracy of approximately 95% for the two datasets. Multiple unrelated studies summarized in this publication also obtained a high level of accuracy in chromosome segmentation and classification. To assess its efficiency, Zhou et al. [39] modified a general-purpose AI image viewer for use with their clinical chromosome image analysis and capture system and achieved a 4.7–6.8-fold efficiency in analysis time for a variety of chromosome abnormalities and specimen types. Although these studies may not fully represent clinical scenarios, they support the applicability of AI-guided karyotyping in the clinical laboratory space.

The implementation of this technology in clinical cytogenetics laboratories is in its infancy. As such, there is little available in the current literature regarding clinical utilization of AI-guided karyotyping, but the early studies have been promising [35,36,40,41,42]. Our laboratory was fortunate to be part of a multicenter clinical laboratory evaluation team for one of the available AI platforms and subsequently incorporated it into our clinical operation for chromosome analysis of blood and bone marrow specimens. Here, we share our validation process and observations regarding the changes and impact of implementing the AI-guided software.

Our validation plan was approached as any other upgrade to instrumentation or software already in use in the laboratory, where the previous platform was compared directly with the new one. The scanning instrumentation and automated (non-AI) version of the analysis software had been in use in our clinical laboratory for several years and had been employed for chromosome analysis of thousands of clinical cases, therefore justifying this approach. We chose to validate the software upgrade using only normal specimens because, at this point, the software is limited in its ability to classify structural chromosome abnormalities; these require manual corrections by the technologist analyzing the case. We were fortunate that, as a benefit of our engagement as a beta testing site, the technologists were already familiar with and trained to use the AI-guided application and no additional training was necessary. Briefly, validation was performed on 300 metaphase cells derived from 15 completed normal (either 46,XX or 46,XY) clinical cases (6 bone marrow and 9 blood specimens) that had been analyzed using the automated algorithm. The slides were re-scanned and analyzed with the AI-guided algorithm. For every case, two technologists, each independently, compared at least ten metaphase spreads and karyograms to those generated by the automated program and assigned an image quality score. The acceptance criteria for the validation were image quality and chromosome placement accuracy at least comparable to those generated by the prior software version. For each case, averaging the 20 assessed cells, the acceptance criteria were met. In addition, improved chromosome contouring, placement accuracy, and time for the AI-guided software to open the karyogram image were observed but not quantified. We plan to add metrics for these criteria for the validation of future upgrades to the software.

**Figure 1:**
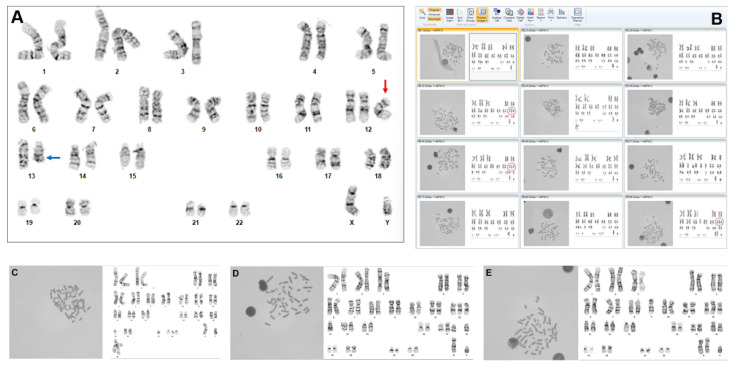
Images from the Applied Spectral Imaging HiBand version 8.4 showing an analysis of a bone marrow specimen from a patient with chronic lymphocytic leukemia. (**A**) Karyogram of a metaphase cell with a karyotype of 47,XY,+12. The image has been reviewed and corrected by a technologist. The red arrow indicates trisomy 12, and the blue arrow indicates a deleted chromosome 13. (**B**) Representative low-definition gallery view showing additional artificial intelligence-generated metaphase-karyogram pairs from the same case prior to review and/or correction. The red circles indicate three copies of chromosome 12 in abnormal cells. (**C**–**E**) Close-up images of three (5th, 7th, and 12th) metaphase–karyogram pairs from the gallery view, enlarged to show detail.

While we are still in the early stages of clinical implementation, we have appreciated ways in which the AI-guided program features could change some of the laboratory’s routine clinical practice. Our basic laboratory practice for specimen processing did not change, nor did the network security, since, in our laboratory, the product resides on the internal medical center-provided servers and is regulated by the virtual environment of the institution. However, as expected based on our beta testing experience, there was an approximately 50% reduction in time required for the analysis of a routine blood or bone marrow case. This occurred for several reasons. Prior to using the AI-guided technology, the automated system required the technologists to wait for the metaphases from the scanner to manually select cells and then wait for each karyogram to load. With AI, processing occurs in real time, and the software presents hundreds of metaphases and their preliminary karyotyped images for review by the technologist. Instead of just scanning for metaphase morphology, the technologists can now assess all the karyograms in the gallery of images for any potential abnormalities. For chromosomally normal cases, the technologist can rapidly review and correct 20 karyograms to complete the case. If an abnormality is identified, the cell can be reviewed and the image corrected as needed (Figure 1A). Then, it is easy (even with less than 100% karyotyping accuracy) to quickly scan for additional cells with a given or related abnormality to establish clonality (Figure 1B–E). Of note, any structural abnormalities captured in the images typically must be manually identified and placement-corrected, since this capability is limited in the implemented product.

There has been an improved diagnostic yield whereby AI has had a surprising impact on the discovery of abnormalities due to the ease it provides for scanning hundreds of karyograms. In our experience, there were multiple instances where a rare abnormal cell visually ”jumped out” because it was AI-karyotyped but would likely have been missed in the previous workflow where metaphases were scanned, but only 20 cells were karyotyped and analyzed by the technologist per standard guidelines [43]. For the same reason, the AI-guided workflow has had utility in locating an abnormality identified by another method, such as a rearrangement detected by fluorescence in situ hybridization (FISH) or a specific abnormality identified in a previous specimen from a cancer patient. The system also helps investigate clonal evolution quickly, since a technologist can scan the image gallery rather than perform the prior laborious process of manually karyotyping dozens of metaphase cells to find the missing related abnormalities.

We have observed more consistent metaphase choice by the technologists, since the metaphase cells are presorted by spread, banding, and overall appearance chosen by the AI algorithm, but this is difficult to quantify. There appears to be less selection bias by the technologists in choosing or avoiding particular metaphase cell morphology for analysis. Of note, this does not apply in cases where it is necessary to manually karyotype metaphases that the software deems too complex for computational analysis. Overall, the ability to scan the presorted metaphase–karyogram paired image gallery has been beneficial in aiding with the identification of cells with chromosome abnormalities.

In contrast, we have also observed inherent biases in the AI-guided system implemented in our laboratory, but this may be specific to each algorithm and laboratory. In our experience, AI-guided karyotyping is the most accurate function and requires the fewest manual corrections for chromosomes in the 400–550 band resolution range. There is limited accuracy classifying complex structural rearrangements, chromosomes with split chromatids, and metaphase cells with multiple overlapping chromosomes which form clusters. These issues will likely be resolved with the on-going training of algorithms, improving more diverse datasets such as medial axis identification and normalization of chromosome features for high-resolution banding patterns.

## 5. Reflections on Experience with AI-Guided Karyotyping

Changes brought about by clinical implementation of AI-guided karyotyping are positive in our experience but have brought issues to the forefront that will need to be addressed moving forward. While development and implementation of AI in karyotyping and other medical diagnostics are still in the early stages, there are practical, regulatory, and ethical challenges that must be considered and addressed [2] as this technology evolves. For example, special attention is needed regarding patient data security and confidentiality with remote use of this software, particularly in the context of products with cloud-based access.

The altered workflow made possible with a gallery of pre-karyotyped cells increases the denominator in defining a clone. Typically, a clonal or mosaic abnormality is defined as the presence of at least 2 cells with the same chromosome gain or structural rearrangement, or at least 3 cells with loss of the same chromosome, out of 20 cells plus up to 30 more if needed [20,43]. However, when scanning through hundreds of AI-guided karyograms to identify additional cells with the targeted abnormality, increasing the number of karyograms may alter the threshold to achieve clonality. On the one hand, if the ratio of cells containing the clonal abnormality to total cells helps define what is a clone, this number is significantly altered; on the other hand, the clinical utility of identifying a rare clone may be of greater importance. This consideration is applicable to both somatic and constitutional cytogenetic analysis.

The quantity of available AI-karyotyped images presents issues of its own. Effective use of technologist time needs to be reassessed and prioritized when there are hundreds of karyograms available for initial review, such as in cases with a good mitotic index. While thorough case evaluation is essential, looking through hundreds of metaphase–karyogram image pairs before starting analysis may not be time-efficient. Guidelines should be developed and vetted, taking into consideration the specimen type, clinical diagnosis, patient medical history, and other factors. Additionally, the current archival practice for images is variable but generally includes at least two for each clonal or mosaic cell line and may include all cells analyzed and/or karyotyped for each patient case [43]. This becomes impractical as the number of AI-karyotyped, technologist-reviewed images expands. This problem is twofold: it involves the number of images archived and the limitations of data storage due to file size. Also, as with all medical records, data security and patient confidentiality are paramount and must be ensured [44].

As AI-guided karyotyping and other AI applications become more commonplace in clinical use, there is a risk for training diminution [4]. Overreliance on technology is often accompanied by reduced hands-on training and grounding of trainees in fundamental concepts. With respect to AI use in clinical cytogenetics, this would result in the loss of training opportunities and impact both the technologist workforce and doctoral-level cytogeneticists. There is, however, an opportunity to incorporate the AI process as a valuable training aid to conserve crucial cytogenetic skills and as part of a comprehensive program that includes the use of other AI tools. For example, after the trainee has mastered basic karyotyping skills, they could be presented with complex practice cases that have been processed with the AI-guided application. These cases would have hundreds of metaphase–karyogram pairs for the trainee to review and correct, greatly expanding the hands-on learning experience currently provided with non-AI systems.

In terms of the workforce, there may be concerns about AI replacing jobs [45], despite an increasing shortage of trained cytogenetic personnel [25]. On the contrary, our experience with AI karyotyping reaffirms the need for skilled technologists, as the technology allows them to focus on higher level tasks such as case analysis and correct nomenclature. Human expertise remains essential for validating and interpreting AI-generated results, especially in complex cases. AI serves as a powerful tool that enhances the capabilities of technologists, allowing them to focus on more critical aspects of their work.

There may be a general concern or misperception regarding the AI-guided karyotyping algorithm evolving continuously while in use in the clinical laboratory. In actuality, these applications operate within the boundaries of the current models, where a static version of the program is used while the technology itself continues to evolve independently. Collaboration between the commercial developers of these AI-guided products and the clinical laboratory end users will continue to be essential to this process. As newer iterations of the software with algorithmic improvements are released, they are integrated into use following clinical validation, creating a continuous loop of application and innovation. Depending on the nature and extent of the updates in each new version, there is a requirement for various degrees of validation and possible user training, like most laboratory technology upgrades.

As AI continues to evolve, it is expected to further streamline processes, reduce turnaround times, and improve the accuracy of cytogenetic analysis. Innovations might include expanding AI pattern recognition to identify complex abnormalities more definitively than is possible with the human eye. The AI algorithm may provide suggested karyotype nomenclature and collate this with nomenclature-describing results generated by other genetic abnormality detection platforms, such as FISH, chromosome microarray, optical genome mapping, and/or next generation sequencing to provide easy access for cross-referenced analysis. Integration with the patient EMR and external reference sources could supply personalized information regarding the clinical significance and prognosis of abnormalities for use by laboratory scientists for result interpretation and reporting. With ongoing advances, such as those suggested here, careful consideration must be given to patient privacy, professional expertise, and regulatory and ethical challenges that accompany these advances.

## 6. Conclusions

In the era of AI and its integration into the practice of laboratory medicine, the incorporation of AI-guided karyotyping in clinical cytogenetics is becoming transformative in the way chromosome analysis is performed. Researchers and early clinical users alike report high accuracy and significant time savings accorded by the use of AI-guided algorithms for the labor-intensive process of identifying and placing chromosomes [3,35,36,39,40,41,42]. Although this technology is still relatively new, the volume and instantaneous availability of data it provides has already altered the paradigm for chromosome analysis. As improvements through continued DNN learning and integration of other clinical, laboratory, and database information occur, updated professional practice and guidance will likely be required. Additionally, it will be critical to maintain a collaborative relationship whereby the AI-guided karyotyping and adjacent technologies serve only as tools, and not a replacement, for professionals, providing high-quality diagnostic service. In this context, the use of AI algorithms provides an innovative and effective pathway to guide the future of clinical cytogenetics.

## Figures and Tables

**Table 1:** Comparison of general features for four commercially availableartificial intelligence-guided karyotyping platforms.

System Feature	ASI [26,27]	BioView [28,29]	Diagens [30,31]	MetaSystems [32,33,34]
Compatible banding techniques	G, Q, R	G	G, R	G, Q, R
Supported specimen types	BL, BM	BL, BM, AM	BL, AM	BL, BM, AM, CV
Scans different specimen types in the same session	Yes	Yes	INA	Yes
Manual and scanning platform integration	Yes	Yes	INA	Yes
Real time karyotype correction tools	Yes	Yes	Yes	Yes
High-definition imaging	Yes	Yes	Yes	Yes
Can use third-party metaphase images	Yes	No	No	No
Abnormality identification algorithm	Yes	Yes	Yes	Yes
Provides ISCN suggestions	Limited	Yes	INA	Yes
Integration with LIS and EMR possible	Yes	Yes	INA	Yes
Remote access	Web	Web, cloud	Cloud	Web
Linked to online database(s) for clinical interpretation	No	No	No	No

ASI, Applied Spectral Imaging; G, Giemsa; Q, quinacrine, R, reverse; BL, blood; BM, bone marrow; AM, amniotic fluid; CV, chorionic villi; INA, information not available; ISCN, international system for cytogenetic nomenclature; LIS, laboratory information system; EMR, electronic medical record.

**Table 2:** Comparison of normal metaphases from blood and bone marrow specimens analyzed with the Applied Spectral Imaging HiBand v8.4 and MetaSystems Ikaros artificial intelligence-guided karyotyping systems.

Metric	ASI [35,36]		MetaSystems [37]	
	Blood	BM	Blood	BM
Number of cases	10	18	45	45
Number of metaphase cells	200	347	900	900
Average analysis time per metaphase	1.6 m	1.7 m	2.8 m	3.0 m
Average analysis time per case (20 metaphases)	32 m	34 m	55.1 m	60.6 m
Average time reduction per case (%), relative to previous automated method	53%	46%	53.9%	42.2%

ASI, Applied Spectral Imaging; BM, bone marrow; m, minutes.

## Data Availability

No new data were created or analyzed in this study. Data sharing is not applicable to this article.
